# Assessing variations in estimates of drowning mortality in Turkey from 2013 to 2019

**DOI:** 10.1186/s13690-022-00944-w

**Published:** 2022-08-01

**Authors:** Ali Işın, Amy E. Peden

**Affiliations:** 1 Department of Coaching Education, Faculty of Sport Sciences, Akdeniz University, Antalya, Turkey; 2 Institute of Health Sciences, Akdeniz University, Antalya, Turkey; 3 School of Population Health, UNSW Sydney, Sydney, NSW 2052 Australia; 4 College of Public Health, Medical and Veterinary Sciences, James Cook University, Townsville, QLD 4811 Australia

**Keywords:** Drowning, Epidemiology, Cause-specific mortality, Causes of Death, Public Health

## Abstract

**Introduction:**

Drowning is an under-recognised public health threat and a leading cause of injury-related mortality and morbidity. However, in many countries, including Turkey, limited data impair understanding of drowning burden and Global Burden of Disease (GBD) Study drowning estimates (defined using International Classification of Diseases [ICD] codes W65–74) do not include flood-related deaths (X38) and water transportation related drownings (V90, V92). A lack of accessible and reliable country-level data impacts a country’s ability to develop appropriate drowning prevention interventions and measure efficacy. This retrospective population-based study aimed to explore differences between two datasets in fatal drowning in Turkey between 2013 and 2019.

**Methods:**

National, all-age data on fatal drownings (restrictive definition: ICD-10 codes W65–74) were sourced from the Turkish Statistical Institute (TurkStat) and the Global Burden of Disease (GBD) study. In addition, a broader definition of drowning including water transport, flood-related deaths and drowning due to undetermined intent (ICD-10 codes W65–74, V90, V92, X38, Y21, T751) were sourced from TurkStat. Numeric and percentage differences in number of drowning deaths were calculated overall and by sex, age group and death year. Chi square (*p* < 0.05) and relative risk (95% confidence intervals) using crude drowning rates per 100,000 population were also calculated for TurkStat data.

**Results:**

From 2013 to 2019, TurkStat reported a total of 5004 drowning deaths (coded W65–74) were reported, compared to 5252 (5% difference; *n* = 248) using the broader definition. A restrictive definition underreported drowning most significantly in females (9.5%; *n* = 97), 5–9 year-olds (8.9%; *n* = 31) and in the 2015 calendar year (30.2%; *n* = 226). Males accounted for 78.8% of drowning in Turkey, with females significantly (*p* < 0.001) more at risk under 10 years of age (0–4 years X^2^ = 67.9; 5–9 years X^2^ = 23.9) and aged 65+ years (X^2^ = 29.7). GBD data overestimated a restrictive definition of drowning by 3.2% overall (7.6% for females, 52.5% for 0–4 year-olds) and underreported drowning for 65+ year-olds by 17% when compared to TurkStat restrictive definition of drowning.

**Conclusions:**

Although a restrictive definition of drowning doesn’t greatly impact estimates at a population level in Turkey, there are variations. This highlights the importance of accurate country-level drowning data to guide decision making for prevention.

## Introduction

Premature mortality is an important indicator of a society’s health and wellbeing [32]. Reliable data on causes of premature mortality, such as injury, are vital to identify who is at greatest risk to guide preventive efforts, including priority setting, economic investment, and policy change [2], as well as a providing a means of identifying emerging threats or evaluating impacts of preventive interventions [31]. In the case of drowning, a global public health concern [7, 33], the World Health Organization (WHO) recommends data collection and well-designed studies as one strategy to prevent drowning [35]. Data are also vital to underpin the development of a national water safety plan, another recommended strategy.

Drowning is the third leading cause of unintentional injury-related death worldwide, after road injury and falls [37]. In 2019, epidemiological data estimated drowning claimed the lives of 236,000 people worldwide [36] however, global estimates are incomplete. Estimates of drowning employed by both the WHO and the Global Burden of Disease (GBD) Study define drowning using the International Classification of Diseases (ICD)-10 codes [34] W65–74 (accidental drowning and submersion). These estimates exclude drowning fatalities due to transportation-related incidents (i.e., boats and watercraft) (V90, V92), victims of flood (X38) and drowning due to undetermined intent (Y21). The inclusion of T751 (a diagnostic code for unspecified effects of drowning and nonfatal submersion) contributes to further capturing all drowning-related deaths. Such a narrow definition of drowning, that is using W65–74 only, is estimated to underreport drowning by between 40 and 60% in high income countries such as Australia, Finland and the United States [14, 17, 18].

Data challenges in the investigation of drowning also persist in Turkey, where drowning is a major public health problem [12, 26]. Official data (the death notification system of the General Directorate of Public Health) has previously been used to explore injury-related mortality (including drowning) in Turkey, however this examination was restricted to children under-five years of age [3]. Similarly, retrospective reviews of hospital records of drowning cases in Turkey have been conducted, however these have also been limited to a single site or hospital or a specific age group [5, 8, 23].

To date, national studies of drowning in Turkey have been conducted using media reports [11, 24, 25]. The use of media data is considered useful for collecting additional variables for the prevention of drowning deaths which are often not available in death data, such as location of incident and activity prior to drowning and is an approach that has been used in several countries, to supplement data from official sources or in the absence of official data [1, 19, 38].

To our knowledge, no previous study has investigated all-age drowning at the national level in Turkey using official sources [3] nor explored the impact of a broader definition of drowning on estimates. Therefore, this study had several aims: 1) To examine drowning fatalities in Turkey by sex, age group and year of death using official data; 2) To compare restrictive (W65–74) and broader definitions of drowning (W65–74, V90, V92, X38, Y21, T751) using ICD-10 codes in Turkey; and 3) To compare official data and GBD study modelled data for drowning for Turkey to identify differences in estimates.

## Materials and methods

This is a total population, retrospective analysis of unintentional fatal drowning which occurred between January 1, 2013 and December 31, 2019 of residents of Turkey.

### Study setting

This study was conducted in Turkey, which has an average population of 80 million between 2013 and 2019. Turkey has 3% of its area in Europe and the rest in Asia and is a bridge between the two continents. Turkey has four seas: the Mediterranean Sea, the Aegean Sea, the Black Sea and the Sea of Marmara, and in addition, Turkey is rich in freshwater with many lakes, dams and rivers. The land area of Turkey is about 780,000 km^2^ [6].

### Data sources

Data were sourced from the Turkish Statistical Institute (TurkStat) and the Institute of Health Metrics and Evaluation’s GBD Study.

### TurkStat data

Data on drowning fatalities of residents of Turkey used in the study were requested from the TurkStat Cause of Death Statistics (2013–2017) and TurkStat Death and Cause of Death Statistics (2018–2019) [29]. Data were sourced based on underlying cause of death. TurkStat gathers death statistics from The Ministry of Interior, General Directorate of Civil Registration and Nationality, the Central Civil Registration System (MERNIS), and the Turkish Statistical Institute Death Reporting System (TurkStat-DRS). The process has been previously described in detail [16]. However, in brief, MERNIS records death events for the whole country. In urban areas, health practitioners certify cause of death. In rural areas without health practitioners, deaths are registered by village headman with lay reported cause of death [16]. TurkStat-DRS covers only urban areas and uses information on the TURKSTAT Death Certificate which records cause of death and is completed by an attending physician if a death occurs in a health facility, by municipality or primary health care physicians if the death occurs at home [16]. Since 2009, TurkStat data includes al reported deaths by merging the MERNIS data as well as data from death certificates submitted by the Provincial Health Directorates to TurkStat [16]. Finally, TurkStat uses the ICD-10 to classify and code cause-of-death [27].

Cases with an underlying cause of death coded to ICD-10 codes of W65–74 were initially requested. This request was then expanded to include both W65–74 as well as deaths coded as X38, T751, V90, V92, or Y21. See Table 1 for an explanation of included ICD-10 codes (Table 1).

**Table 1:** International Classification of Diseases (ICD)-10 drowning-related codes and definition

ICD-10 codes	Definition /explanation
W65	Drowning and submersion while in bath-tub (Accidental)
W66	Drowning and submersion following fall into bath-tub (Accidental)
W67	Drowning and submersion while in swimming-pool (Accidental)
W68	Drowning and submersion following fall into swimming-pool (Accidental)
W69	Drowning and submersion while in natural water (Accidental)
W70	Drowning and submersion following fall into natural water (Accidental)
W73	Other specified drowning and submersion (Accidental)
W74	Unspecified drowning and submersion (Accidental)
X38	Victim of flood
T75.1	Drowning and nonfatal submersion
V90	Accident to watercraft causing drowning and submersion
V92	Water-transport-related drowning and submersion without accident to watercraft
Y21	Drowning and submersion, undetermined intent

*Note*: *ICD-10* International Classification of Diseases (ICD) 10th Revision

Both data sets were requested by sex, age group and year of death for the period 2013–2019. Since TurkStat presents death and population data in five-year age groups (up to 65+ years) in accordance with the World Health Organization (WHO), the age groups in this study were divided and evaluated into 14 groups from 0 to 4 years, through to 65+ years.

### GBD Study data

Data were sourced for cause of death C2.2 Drowning (defined as ICD-10 codes W65–74) for Turkey between 2013 and 2019 from the Institute of Health Metrics and Evaluation GBD Results Tool [9]. Data input sources for modelling drowning as a cause of death in Turkey comprise several academic studies, verbal autopsy surveys and Turkey Vital Registration deaths data from 2009 to 2016 [10]. Data were sourced by total, by sex, and by five-year age groups from 0 to 4 years to 65+ years to match the TurkStat data. GBD data were rounded up to whole numbers to match the presentation of the TurkStat data.

### Data coding and analysis

Data on drowning deaths were presented as frequencies and percentages by age group, sex and year of incident. For TurkStat data, chi square (χ^2^) analysis (statistical significance *p* < 0.05) was undertaken to explore differences between males and females in fatal drowning in Turkey. In addition, the crude mortality rate (per 100,000 population), was calculated using population data obtained from TurkStat population and demographic statistics [28]. Rates were also used to calculate the relative risk (RR) of fatal drowning, with a 95% confidence interval (CI). The group with the lowest rate was considered as the reference group.

To compare between restrictive and broader estimates of drowning, the difference between the two was calculated as a percentage of the number of restrictive definition drowning fatalities. For the comparison between TurkStat and GBD estimates, the difference between the two figures (firstly TurkStat restrictive definition vs GBD estimate and secondly TurkStat broader definition vs GBD estimate) was calculated as a percentage of the TurkStat number of cases. This was conducted overall and by sex, age group and year of drowning incident.

### Ethics

This study forms part of a broader research collaboration exploring drowning in Turkey [12], for which human research ethics approval has been received (University of New South Wales Human Research Ethics Committee [HC210244]). This research observes the Guidelines for Accurate and Transparent Health Estimates Reporting [22] and is conducted in compliance with Helsinki Principles and the European Union and National Laws.

## Results

Between 2013 and 2019, a total of 5004 drowning fatalities were recorded by TurkStat using the restrictive definition of W65–74. This varied by 248 (or 5.0%) when additional ICD codes were included, comprising a total of 5252 drowning deaths across the same period. When examined by sex, the underreport is greater among females, with a difference of 9.5% between the two definitions of drowning, compared to 3.8% for males.

The underreport is greatest among those of unknown age (14.8%; *n* = 12) followed by 40–44 year-olds (7.7%; *n* = 16) and 0–4 year-olds (7.5%; *n* = 33). The smallest differential was seen among people aged 65+ years (0.7%; *n* = 6). When comparing drowning by year of incident, the year 2015 reports the biggest differential, a difference of 30.2% or 226 deaths between the restrictive and broader definitions of drowning. (Table 2).

**Table 2:** Difference between W65–74 and broader definition of drowning using TurkStat data, Turkey, 2013–2019

	Restrictive drowning definition	Broader drowning definition	% difference	N difference
**Total**	5004	5252	5.0	248
**Sex**				
**Male**	3985	4136	3.8	151
**Female**	1019	1116	9.5	97
**Age Group (in years)**				
0–4	442	475	7.5	33
5–9	348	379	8.9	31
10–14	414	433	4.6	19
15–19	621	639	2.9	18
20–24	445	467	4.9	22
25–29	360	382	6.1	22
30–34	278	293	5.4	15
35–39	238	248	4.2	10
40–44	207	223	7.7	16
45–49	185	196	5.9	11
50–54	192	204	6.3	12
55–59	206	213	3.4	7
60–64	194	199	2.6	5
65+	802	808	0.7	6
Unknown age	81	93	14.8	12
**Year of death**				
2013	691	694	0.4	3
2014	762	767	0.7	5
2015	749	975	30.2	226
2016	911	920	1.0	9
2017	758	759	0.1	1
2018	601	602	0.2	1
2019	532	535	0.6	3

*Note*: restrictive definition = ICD codes W65–74. Broader definition = ICD codes W65–74, V90, V92, X38, Y21 and T751. Numerical (N) and percentage (%) difference between the restrictive definition of drowning and the broader definition of drowning

**Fig. 1:**
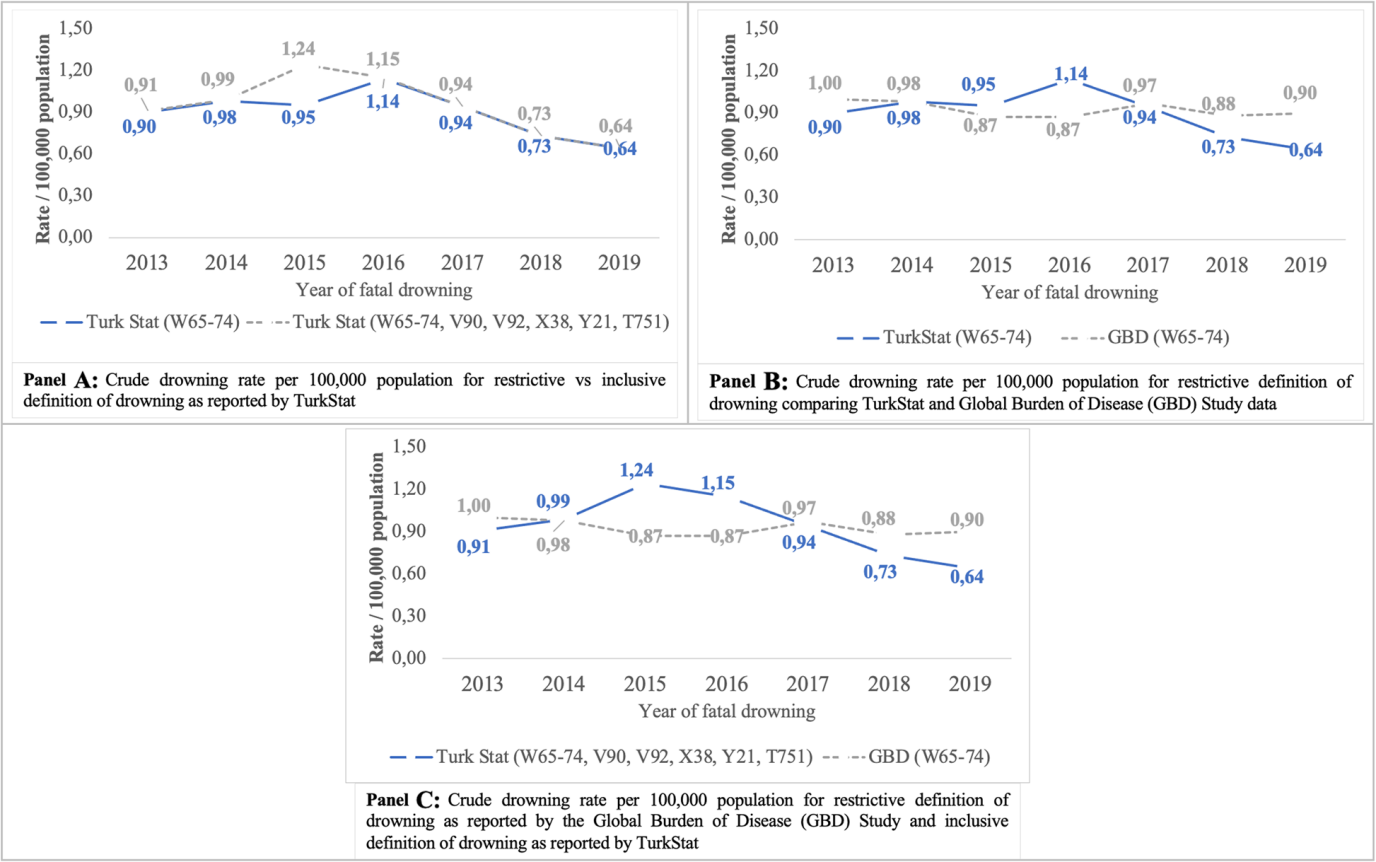
Crude fatal drowning rates per 100,000 resident population, Turkey, 2013–2019

When computed as rates per 100,000 population, the greatest differential can be seen in 2015, with a restrictive definition of drowning resulting in a fatal drowning rate of 0.95 per 100,000 population, compared to a rate of 1.24 per 100,000 when a more inclusive definition is used (Fig. 1; Panel A).

Of the 5252 drowning deaths which occurred between 2013 and 2019, 78.8% (*n* = 4136) were males. When exploring differences in fatal drowning comparing males and females, females were significantly more likely to drown than males if aged 0–4 years (χ^2^ = 67.9; *p* < 0.001), 5–9 years (χ^2^ = 23.9; p < 0.001) or 65+ years (χ^2^ = 29.7; p < 0.001). Fatal drowning risk was significantly higher for males aged 15–19 years (χ^2^ = 40.6; p < 0.001), 20–24 years (χ^2^ = 23.9; p < 0.001), and 25–29 years (χ^2^ = 14.4; p < 0.001) when compared to females. (Table 3).

**Table 3:** Absolute number and percentage of unintentional drowning deaths (broader definition) by sex and age group using TurkStat data, chi square (χ^2^) comparing males and females, Turkey 2013–2019

	Total		Male		Female		χ^**2**^ comparing males to females (***p*** value)
	n	%	n	%	n	%	
**Total**	5252	100.0	4136	78.8	1116	21.2	1736.558 (p < 0.001)
**Age group (in years)**							
0–4	475	9.0	304	64.0	171	36.0	67.906 (p < 0.001)
5–9	379	7.2	261	68.9	118	31.1	23.855 (p < 0.001)
10–14	433	8.2	334	77.1	99	22.9	0.735 (*p* = 0.391)
15–19	639	12.2	565	88.4	74	11.6	40.641 (p < 0.001)
20–24	467	8.9	409	87.6	58	12.4	23.879 (p < 0.001)
25–29	382	7.3	330	86.4	52	13.6	14.357 (p < 0.001)
30–34	293	5.6	248	84.6	45	15.4	6.435 (*p* = 0.112)
35–39	248	4.7	204	82.3	44	17.7	1.913 (*p* = 0.167)
40–44	223	4.2	172	77.1	51	22.9	0.366 (*p* = 0.545)
45–49	196	3.7	161	82.1	35	17.9	1.400 (*p* = 0.237)
50–54	204	3.9	164	80.4	40	19.6	0.342 (*p* = 0.559)
55–59	213	4.1	171	80.3	42	19.7	0.311 (*p* = 0.577)
60–64	199	3.8	157	78.9	42	21.1	0.003 (*p* = 0.960)
65+	808	15.4	578	71.5	230	28.5	29.716 (p < 0.001)
Unknown age	93	1.8	78	83.9	15	16.1	1.483 (*p* = 0.223)

*Note*: n = number and % = percentage; χ^2^ = Chi-squared test

**Fig. 2:**
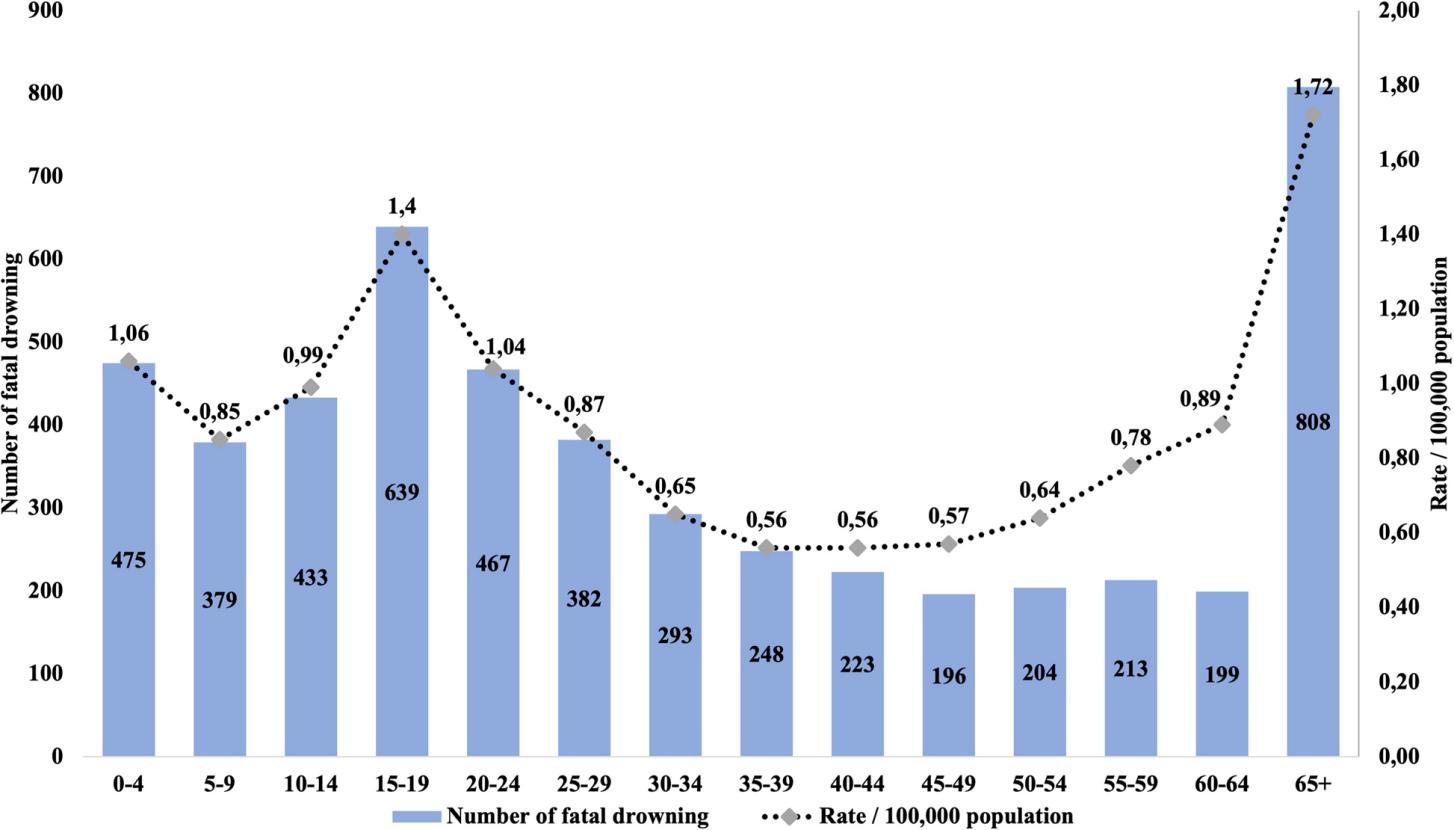
Number of fatal drownings by age group and crude drowning mortality rate per 100,000 population, Turkey, 2013–2019

**Fig. 3:**
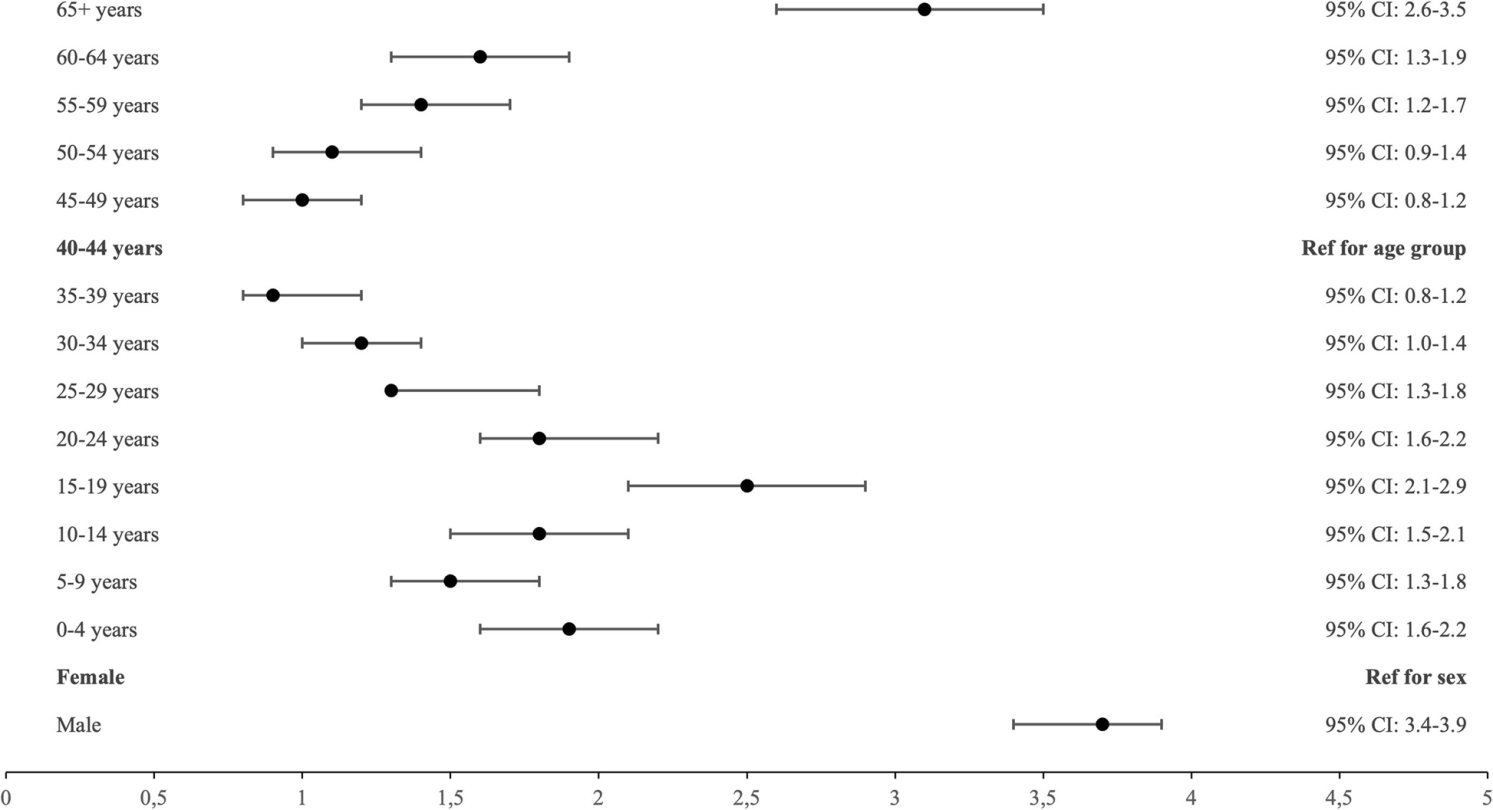
Relative risk (RR) (95% confidence interval) of drowning deaths (broader definition) by sex and age group using TurkStat data, Turkey 2013–2019 (Where RR was calculated, the group with the lowest rate was used as the reference point (The reference point; female for sex and 40–44 years for age group)

Using the broader definition of drowning, fatal drowning rates peaked among those aged 15–19 years (1.47 per 100,000 population) and was lowest among those aged 65+ years (0.66 per 100,000) (Fig. 2). The unintentional drowning mortality rate for males was 1.47/100000 during the study period, higher than the rate for females (0.40/100,000). Males were 3.7 times (95% CI:3.4–3.9) more likely to unintentionally drown than females. Compared to the 40–44 age group, those aged 65 years and over had 3.1 times (95% CI: 2.6–3.5) greater risk of drowning (Fig. 3).

When comparing TurkStat cause of death data to GBD study modelled estimates for the more restrictive definition of drowning, GBD data reports 3.2% more drowning deaths overall than TurkStat. The differential between the two sources is most pronounced for females with GBD estimated 7.6% more drowning deaths among females than TurkStat. The differential is greatest among the younger age groups, with the GBD study reporting 52.5% more drowning deaths than TurkStat for 0–4 year-olds, 29.6% more deaths for 5–9 year-olds and 18.6% more deaths for 10–14 year-olds. The inverse is true among older age groups with the GBD study reporting 17.0% less drowning deaths for people aged 65 years and over than TurkStat data. GBD study data has moved from underreporting drowning in Turkey by 13.9% in 2016, to reporting 30.5% more deaths than TurkStat for 2019 (Table 4). As a rate per 100,000, the 2016 differential moves from a TurkStat reported crude fatal drowning rate of 1.14, compared to the GBD study reported fatal drowning rate of 0.87. In 2019, this moves from a fatal drowning rate of 0.64 per 100,000 population as per TurkStat data, to a rate of 0.90 per 100,000 for GBD study data. (Fig. 1; Panel B).

**Table 4:** Comparison of TurkStat data to GBD modelled estimates for drowning, Turkey, 2013–2019

	Restrictive drowning definition			Broader drowning definition	Restrictive drowning definition	% difference
	TurkStat	GBD	% difference	TurkStat	GBD (W65–74 only)	
	n	n	%	n	n	%
**Total**	5004	5164	3.2	5252	5164	−1.7
**Sex**						
Male	3985	4068	2.1	4136	4068	−1.6
Female	1019	1096	7.6	1116	1096	−1.8
**Age group (in years)**						
0–4	442	674	52.5	475	674	41.9
5–9	348	451	29.6	379	451	19.0
10–14	414	491	18.6	433	491	13.4
15–19	621	583	−6.1	639	583	−8.8
20–24	445	452	1.6	467	452	−3.2
25–29	360	376	4.4	382	376	−1.6
30–34	278	277	−0.4	293	277	−5.5
35–39	238	243	2.1	248	243	−2.0
40–44	207	211	1.9	223	211	−5.4
45–49	185	177	−4.3	196	177	−9.7
50–54	192	194	1.0	204	194	−4.9
55–59	206	187	−9.2	213	187	−12.2
60–64	194	184	−5.2	199	184	−7.5
65+	802	666	−17.0	808	666	−17.6
Unknown	81	–	–	93	–	–
**Year of death**						
2013	691	686	−0.7	694	686	−1.2
2014	762	752	−1.3	767	752	−2.0
2015	749	766	2.3	975	766	−21.4
2016	911	784	−13.9	920	784	−14.8
2017	758	759	0.1	759	759	0.0
2018	601	722	20.1	602	722	19.9
2019	532	694	30.5	535	694	29.7

Please note: GBD estimates may not internally sum between total and age groupings for example due to rounding and/or modelling. Restrictive definition = ICD codes W65–74. Broader definition = ICD codes W65–74, V90, V92, X38, Y21 and T751. TurkStat: Turkish Statistical Institute. GBD: Global Burden of Disease. N = number and % = percentage

When examining differences between the two data sets using the broader definition of drowning from TurkStat data, GBD study data slightly underreports drowning overall (− 1.7%) and for both sexes (− 1.6% for males and − 1.8% for females). By age group, drowning among 0–4 (+ 41.9%), 5–9 (+ 19.0%) and 10–14 (+ 13.4%) year-olds remain overestimated compared to TurkStat data. Drowning among 65+ year-olds is slightly more underreported (− 17.6%) with the inclusion of additional drowning-related codes. Drowning is most significantly underreported by the GBD study in 2015 when the broader definition of drowning is used, but remains overestimated in 2018 and 2019, even with the inclusion of additional drowning codes (Table 4). The variations across the two data sources and methods, quantified as crude fatal drowning rates per 100,000, are depicted in Fig. 1, Panel C.

## Discussion

Drowning is a leading cause of injury-related death worldwide, yet indicators of global burden are incomplete [14, 17, 18]. In addition, a lack of data at a country-level hampers the ability of downing prevention researchers, advocates and policy makers to identify risk factors and develop both interventions and a National Water Safety Plan [35].

This study is the first population level study of drowning in Turkey using official sources and the first to compare estimates between data sources and coding methodologies. This study identifies the importance of accurate and timely death registration, something which has been a challenge in Turkey [16]. With the known weaknesses of death registration, modelled data, such as that within the GBD study, has an important role to play.

So too does media reporting of drowning [1, 19, 38]. The data from both TurkStat and the GBD analysed in this study, are limited to age group, sex, and year of incident. No further detail is provided to aid in risk factor identification or the development of prevention interventions, such as body of water, activity prior to drowning, impact of alcohol, presence of lifejackets and pre-existing medical conditions, among others. Studies analysing drowning in Turkey via media reports have identified freshwater such as rivers and lakes to be the location of most concern for drowning in Turkey [12, 25], detail which is not available in either of the data sources analysed in this study.

With respect to estimates, there is a growing belief that drowning deaths due to transport and disaster should be included in official estimates given the impact of such deaths in both high-income and low-and middle-income countries [14, 17, 18]. The present study found only a 5% difference between the restrictive and broader definitions of drowning in Turkey between 2013 and 2019, however there were important age and sex-based variations. A broader definition of drowning appears to capture more female drowning deaths (9.5% difference, compared to 3.8% for males) and drowning deaths among 5–9 year-olds (8.9% difference).

Additionally, this study identified 30.2% more drowning deaths in 2015 using the broader definition of drowning which requires further exploration. One possible explanation is the reported 229 individual flood events in 2015 in Turkey may have been more deadly than previous years however, there were a further 331 flood events recorded in 2018 and 332 in 2019, which have not corresponded to an increase in deaths in those years when the broader definition of drowning was used [15].. Further research is required into the causes of death during floods in Turkey, as well as the impact of boating incidents on Turkish residents in 2015. Taking a broader definition of drowning allows for the inclusion of additional ministries and policy approaches to reducing drowning risk, thus amplifying multi-sectoral action [35]. These include disaster preparedness, mitigation, and response strategies such as early warning systems, evacuation strategies and appropriately trained first responders, as well as boating regulation such as vessel construction standards, lifejacket wear, weather alert systems and proper loading of vessels [21].

When drowning deaths were examined by age group, there were large differences seen between the number of registered drowning deaths in children aged 0–4 years in TurkStat data and GBD study modelled estimates, with GBD data reporting 52.5% more drowning deaths in this age group. Known challenges exist in death reporting in Turkey, in particular for infants [16] and therefore, modelled data for drowning among children under five may be more accurate than registered deaths. If policy makers were to consider the size of the burden of child drowning in Turkey using registered death data only, the issue is likely to be greater than currently realised.

Conversely, drowning deaths among the 65+ years age group in Turkey are underreported by 17% in GBD data when compared to registered death data held by TurkStat. Drowning deaths among older people have been described as hidden epidemic [4] suffering a lack of attention in comparison to child drowning. Given a globally ageing population, and people aged 65+ recording the highest rate of fatal drowning in Turkey, there is a need for policy and preventive focus on reducing drowning risk among this age group [20]. Future research of drowning among this age group, should seek to explore risk differences within this broad age group.

With accurate, timely and reliable drowning data comes the ability to develop a National Water Safety Plan to reduce drowning in Turkey [35]. The recent UN Declaration on Global Drowning Prevention [30], of which Turkey is a signatory, also presents an opportunity for advocates and researchers to further the goal of drowning reduction in Turkey, but any such ambition must start with accurate, and inclusive, data.

### Strengths and limitations

To the best of our knowledge this is the first population-based study of all-age drowning in Turkey based on official sources. It is also the first to compare restrictive and broader definitions of drowning to identify the impact on drowning estimates, as well as to compare official cause of death data with GBD study modelled drowning data for Turkey. However, there are some limitations associated with this study. Although official cause of death data were sourced from TurkStat, such data have yet to be evaluated for their completeness and validity [16]. Similarly, previous research has indicated significant gaps in recorded demographic information, in particular for infant deaths [16]. Data from both TurkStat and the GBD study are limited and do not provide further information for the identification of additional risk factors beyond age and sex, such as drowning location and activity prior to drowning. TurkStat requirements as data custodian did not allow for the breakdown of the 65+ years age group into smaller age bands, nor were we able to isolate drowning deaths by individual ICD codes. We believe GBD study data refers to deaths of residents of Turkey only, based on the inputs into the modelling for cause of death data for Turkey [10]. However, should there be a different methodology employed with respect to deaths of non-residents between TurkStat and GBD, this will impact comparisons. Caution must be used with the deaths attributed to ICD code X38 – victim of flood, as other causes of death due to floods may be included, although drowning is the leading cause of death during flooding [13].

## Conclusion

This study has explored different data sources and definitions of the burden of fatal drowning in Turkey. Results indicate the currently known burden of fatal drowning in Turkey is 5 % greater when expanding the definition of drowning. With a broader definition of drowning highlighting increased drowning mortality among females and older people. A policy focus on the broader causes of drowning, such as strategies to improve boating safety and improving flood disaster preparedness and response in Turkey will reduce drowning burden. Accurate, timely and detailed data are essential for the development and evaluation of drowning prevention interventions, guided by a National Water Safety Plan.

## Data Availability

TurkStat data are unable to be publicly shared due to rules around data usage. Interested parties may contact TurkStat and make their own data request via email to: info@tuik.gov.tr. GBD data are publicly available via the GBD Results Tool in the Global Health Data Exchange: http://ghdx.healthdata.org/gbd-results-tool
